# Comparison of Two Immunoassay Screening Methods and a LC-MS/MS in Detecting Traditional and Designer Benzodiazepines in Urine

**DOI:** 10.3390/molecules27010112

**Published:** 2021-12-24

**Authors:** Brian Rossi, Francesca Freni, Claudia Vignali, Cristiana Stramesi, Giancarlo Collo, Claudia Carelli, Matteo Moretti, Dario Galatone, Luca Morini

**Affiliations:** Department of Public Health, Experimental and Forensic Medicine, University of Pavia, Via Forlanini 12, 27100 Pavia, Italy; brian.rossi01@universitadipavia.it (B.R.); frafre93@gmail.com (F.F.); claudia.vignali@unipv.it (C.V.); cristiana.stramesi@unipv.it (C.S.); giancarlo.collo@unipv.it (G.C.); claudia.carelli01@universitadipavia.it (C.C.); matteo.moretti19@gmail.com (M.M.); dario.galatone01@universitadipavia.it (D.G.)

**Keywords:** immunoassay, urine, benzodiazepine, designer benzodiazepine, flubromazepam

## Abstract

Sensitive and specific immunoassay screening methods for the detection of benzodiazepines in urine represent an important prerequisite for routine analysis in clinical and forensic toxicology. Moreover, emerging designer benzodiazepines force labs to keep their methodologies updated, in order to evaluate the reliability of the immunochemical techniques. This study aimed at evaluating the sensitivity and specificity of two different immunoassay methods for the detection of benzodiazepines in urine, through a comparison with the results obtained by a newly developed liquid chromatographic tandem mass spectrometric (LC-MS/MS) procedure. A cohort of authentic urine samples (N = 501) were processed, before and after a hydrolysis procedure, through two immunoassays and an LC-MS/MS method. The LC-MS/MS target procedure was optimized for monitoring 25 different molecules, among traditional and designer benzodiazepines, including some metabolites. At least one of the monitored substances was detected in 100 out of the 501 samples. A good specificity was observed for the two immunoassays (>0.99), independently of the cut-offs and the sample hydrolysis. The new kit demonstrated a fairly higher sensitivity, always higher than 0.90; in particular, a high cross-reactivity of the new immunoassay was observed for samples that tested positive for lorazepam and 7-aminoclonazepam. The two immunoassays appeared adequate to monitor not only traditional benzodiazepines but also new designer ones.

## 1. Introduction

The increasing misuse of benzodiazepines has represented an emerging issue in the last years. Indeed, both a diverted consumption of benzodiazepines from the therapeutic use and an increase in the diffusion of molecules related to the benzodiazepine class, but without a license for medical use, have been reported [1]. These drugs are prescribed to treat several pathologies and disorders such as anxiety, insomnia, and seizures; however, the chronic consumption of benzodiazepines may cause addiction. The consumption of benzodiazepines for non-medical purposes is one of the factors involved in the increasing number of deaths and other negative incidences in North America and Europe [2]. Benzodiazepines’ main pharmacological effect is related to their binding to the neurotransmitter gamma-aminobutyric acid (GABA) on the benzodiazepine site of the GABA type A receptor, leading to a central nervous system depression [3]. Benzodiazepine detection in urine is a common routine test in clinical and forensic laboratories. They are monitored in cases of suspected drug-facilitated sexual assault [4,5,6,7,8,9], in workplace contexts [9,10,11], in suspected impaired drivers [12,13,14,15,16], and in cases of suspected intoxication [17,18,19]. Hence, the laboratories need a sensitive immunoassay for use as a screening procedure. To date, different immunoassay techniques have been developed for the identification and semiquantitative determination of benzodiazepines in urine, such as the cloned enzyme donor immunoassay (CEDIA^®^) [20], kinetic interaction of microparticles in solution (KIMS^®^) [21], enzyme-linked immunosorbent assay (ELISA^®^) [22], and enzyme-multiplied immunoassay technique (EMIT^®^) [23]. Additionally, new designer benzodiazepines have been recently tested with different immunoassays to evaluate the reliability of screening methods in identifying new illicit substances emerging in the black market [19]. The cross-reactivity of the developed kits represents the main issue of whether the assay should be applied to a wide class of substance rather than to a specific molecule. In particular, EMIT^®^, CEDIA^®^ and KIMS^®^ techniques provided good results for specific benzodiazepines only; on the contrary, a sensitivity to lorazepam, a benzodiazepine frequently prescribed and detected in urine samples, is generally limited [24,25,26]. Moreover, several benzodiazepines are mainly excreted in urine as glucuronide conjugates. For examples, lorazepam-glucuronide represents 75% of the total, while temazepam and oxazepam are conjugated at 73% and 61%, respectively [24]. Hence, hydrolysis is always needed to achieve an adequate sensitivity. Some immunoassays increased the sensitivity by including β-glucuronidase solution in the kit [25].

The objective of this work is a pre-market study to evaluate the ARK™ HS Benzodiazepine II Assay on the automated analyzer Siemens ADVIA^®^ 1800 Clinical Chemistry System. The results were compared to those obtained through the Siemens EMIT^®^ II PLUS Benzodiazepine Assay. The study focused on the measurement of the sensitivity and specificity of the two available immunoassays. In particular, since a negative result after a screening test will not be processed through a confirmation analysis, it is extremely important to improve the laboratory workflow with new generation immunoassay kits that guarantee elevated performances. The study was carried out on urine samples from the ordinary tested population in the laboratory, alongside the laboratory’s normal routine. Though urine collected from patients under withdrawal treatment are generally analyzed without any pretreatment, all the samples were tested before and after conventional enzymatic hydrolysis with β-glucuronidase from *Escherichia Coli* (37 °C; 12 h). All samples underwent a confirmation test by LC-MS/MS. Data obtained from LC-MS/MS were chosen as the gold standard for the immunoassay performance evaluation. Finally, the cross-reactivity of the two immunoassays for new designer benzodiazepines was tested by processing spiked urine samples.

## 2. Results and Discussion

All 501 collected samples were analyzed, and 100 samples tested positive for at least one of the monitored substances through the LC-MS/MS analysis. Table 1 reports the molecules and the concentrations measured in samples after and before hydrolysis, respectively. Hydrolysis proved to be a necessary sample pretreatment procedure to increase the diagnostic sensitivity. Indeed, oxazepam was detected in 45 hydrolyzed samples, resulting as the most frequently identified molecule, and only in 24 non-hydrolyzed ones, confirming that this benzodiazepine undergoes an extensive glucuronidation [27]. Additionally, lorazepam, delorazepam, desalkylflurazepam, and 2-hydroxyethylflurazepam were mainly excreted as glucuronide conjugates. Conversely, nordazepam, diazepam and alprazolam appeared as benzodiazepines that had a detectability rate in urine that was less influenced by hydrolysis.

LC-MS/MS data have been selected as gold standards to check for the diagnostic sensitivity and specificity of the two immunochemical screenings. The performance has been evaluated at three different cut-offs: 50, 100, and 200 ng/mL.

The sensitivity and specificity were measured in samples before and after the hydrolysis procedure. Table 2 reports the performance of the two immunoassays at different cut-off values. A high rate of specificity was always achieved, independently of the immunoassay and the cut-off value. On the contrary, a higher sensitivity was observed for the newly proposed ARK^TM^ immunoassay, independently of the cut-off and the sample treatment. In particular, by choosing a threshold of 50 ng/mL, no false negatives were observed in hydrolyzed samples with the new immunoassay. A lower sensitivity was instead observed for the routinely used technique, especially at the suggested cut-off of 200 ng/mL (SE: 0.64). Indeed, 36 out of 100 positive cases provided a negative result, thus confirming the relatively elevated number of false negatives obtained by the EMIT^®^ II PLUS Benzodiazepine Assay.

Nine substances have been individually identified at least once in real samples (n = 29). The most frequently detected substance on its own was alprazolam (n = 8); 7-aminoclonazepam and oxazepam were detected in four samples, lorazepam and nordazepam in three, triazolam, bromazepam and delorazepam in two, and flurazepam only in one sample. The cross-reactivity of alprazolam, 7-aminoclonazepam, oxazepam, and lorazepam has been tentatively measured for the two immunoassays, since these substances were individually detected in at least three authentic cases. Except for alprazolam, cross-reactivities were higher for the ARK™ HS Benzodiazepine II Assay. All four tested substances provided a cross-reactivity on the newly proposed kit that was higher than 100%, with the highest value for 7-aminoclonazepam and lorazepam. In particular, the ARK^TM^ immunoassay demonstrated an elevated sensitivity for lorazepam. Conversely, a limited sensitivity for lorazepam was observed on the EMIT^®^ II PLUS immunoassay, with a cross-reactivity lower than 50%. Due to the signal intensity achieved in the urine samples being positive for lorazepam, the sensitivity of the LC-MS/MS method has been improved, by increasing the volume of urine to be extracted (from 100 µL to 300 µL). The five samples that provided a false positive result on the ARK™ HS Benzodiazepine II Assay were re-processed through the improved LC-MS/MS method, confirming the presence of lorazepam in traces (<10 ng/mL) in all of them.

A case of intoxication by flubromazepam was included in the study, and both immunoassays provided positive results, with a signal intensity higher than the calibration range even after a sample dilution (1:100), confirming a good cross-reactivity of the two kits for this designer benzodiazepine. Since among the monitored designer benzodiazepines only flubromazepam was detected in a real case, blank urine samples were spiked with the remaining seven molecules, at the concentration of 200 ng/mL. The samples were evaluated with the two immunoassays, and the obtained cross-reactivity is reported in Table 3. A limited cross-reactivity was observed in the two immunoassays for bentazepam, while diclazepam provided the highest signal. However, this study proved that new designer benzodiazepines could also be detected through immunochemical screening methods.

## 3. Study Drawbacks

The main weakness of the study is represented by the relatively limited number of real samples that were positive for only one molecule. Indeed, except for alprazolam, the other substances were detected individually in few cases, thus preventing any statistically relevant measurements on single molecules. However, only parent drugs and few metabolites have been monitored through the LC-MS/MS analysis; hence, one cannot exclude that the response of the immunoassay depends exclusively on the active substance rather than on other potentially present metabolites. However, the LC-MS/MS method only included 25 molecules, and other traditional or new designer benzodiazepines could be present as well.

## 4. Materials and Methods

### 4.1. Reagents

Diazepam, nordiazepam, delorazepam, 7-aminoclonazepam, 7-aminoflunitrazepam, alprazolam, α-hydroxyalprazolam, 2-hydroxyethylflurazepam, bromazepam, clonazolam, desalkylflurazepam, etizolam, flurazepam, lorazepam, lormetazepam, midazolam, phenazepam, diclazepam, diazepam-D5, 7-aminoclonazepam-D4, and alprazolam-D5 were obtained from Cerilliant Corporation (Merck, Milan, Italy); flualprazolam, flubromazolam, and flubromazepam were purchased from Cayman Chemical Company (LGC standards, Milan, Italy); oxazepam, triazolam, and temazepam salts were purchased from Formenti (Formenti SPA, Milan, Italy); bentazepam methanolic solution, at a concentration of 0.1 mg/mL, was kindly donated from Italian Early Warning System, Italian Institute of Health (ISS).

Formic acid for mass spectrometry was obtained from Merck (Milan, Italy). High-performance liquid chromatography (HPLC) grade methanol and acetonitrile were purchased from Carlo Erba SRL (Milan, Italy). N-Hexane and ethyl acetate were purchased from Merck (Milan, Italy). β-glucuronidase from *Escherichia Coli* was purchased from Merck (Milan, Italy).

### 4.2. Instrumentation

Siemens ADVIA^®^ 1800 Clinical Chemistry System was used for immunoassay kits processing.

The EMIT^®^ II PLUS Benzodiazepine Assay, controls, and calibrators were donated by Siemens (Milan, Italy). The ARK™ HS Benzodiazepine II Assay, controls, and calibrators were donated by ARK Diagnostic, Inc. (Fremont, CA, USA).

The ARK HS Benzodiazepine II Assay system is composed of liquid ready-to-use reagents, calibrators, and quality controls.

- R1 Reagent (Antibody/Substrate Reagent): contains antibodies to benzodiazepine derivative (etizolam) in buffer solution and the enzyme substrate.
- R2 Reagent (Enzyme Reagent): contains benzodiazepine derivative (etizolam) labeled with recombinant glucose-6-phosphate dehydrogenase (rG6PDH).
- Calibrators and Quality Controls: benzodiazepine derivative (etizolam) in human urine matrix.

Liquid chromatographic tandem mass spectrometry (LC-MS/MS) analyses were performed with an Agilent 1290 Series System (Agilent Technologies, Palo Alto, CA, USA) coupled with a 4000 Q-TRAP (AB Sciex, Foster City, CA, USA) with an electrospray (ESI) Turbo V™ Ion Source. The LC instrumentation was composed of a binary pump and an autosampler. The injector needle was externally washed with methanol for 3 s prior to any analysis. A Kinetex C18 column (100 × 2.1 mm i.d., 2.6 µm particle size) (Phenomenex, Castelmaggiore, BO, Italy) was kept at 30 °C during the analysis. The mobile phase consisted of an aqueous solution with 0.1% (*v*/*v*) formic acid (A) and acetonitrile with 0.1% (*v*/*v*) formic acid (B). The chromatographic elution was the following: gradient elution 95% A maintained for 3 min at the flow rate of 0.25 mL/min, then from 95% to 5% within 12 min, maintaining 30% A between 3 and 5 min, and a final re-equilibration step of 4 min at the flow rate of 0.4 mL/min. The ESI source settings were: ion-spray voltage +5000 V, source temperature 400 °C, and nebulization and heating gas (air): 30 psi and 35 psi, respectively. Multiple reaction monitoring (MRM) was optimized using nitrogen as the collision gas (with the pressure set at level 5) and a dwell time of 30 milliseconds, in positive polarization. Two transitions for each substance were chosen for the identification; the most intense was used for quantification purposes. Data acquisition and elaboration were performed by the Analyst software (version 1.5.1, AB SCIEX).

The LC-MS/MS procedure has been fully validated, following the international guidelines [28]. The limits of detection (LOD) and limits of quantification (LOQ) of the procedure, together with the list of monitored substances, is reported in Table 4.

### 4.3. Validation of the ARK^TM^ Immunoassay

#### 4.3.1. Calibration and Quality Control

Multipoint calibration curves (Logit Log 2 formula) were obtained using five calibrators, provided by the manufacturer, containing increasing concentrations of etizolam: 0.0 ng/mL, 100.0 ng/mL, 200.0 ng/mL, 1000.0 ng/mL, and 3000.0 ng/mL. The calibration curves were verified by assaying the ARK Low- and High-quality controls (75.0 ng/mL and 125.0 ng/mL for a 100.0 ng/mL cut-off; 150.0 ng/mL and 250.0 ng/mL for a 200.0 ng/mL cut-off). The instrument performance was assayed each morning before starting any runs by testing the ARK Controls.

Expected Accuracy: the mean of each control should fall within 15% of nominal.

#### 4.3.2. System Check

The purpose of the system check is to verify the acceptable operation of the analyzer, materials, and operator training at the beginning of the study. Two separate runs were carried out, separate calibrations were performed for each run, and the runs were verified using the following controls: 75.0 ng/mL, 100.0 ng/mL, 125.0 ng/mL, 150.0 ng/mL, 200.0 ng/mL, and 250.0 ng/mL of etizolam (20 replicates for each control).

An acceptable precision and accuracy of quality controls must be obtained.

Expected Precision: ≤12% total CV for concentrations ≥ 50 ng/mL or SD <15 for values <50 ng/mL

Expected Accuracy: the mean of each control should preferably fall within 15% of nominal.

#### 4.3.3. Performance Characterization

##### Precision

A calibration curve was performed, and quality controls were tested. Two runs a day were carried out over a 5-day protocol.

Run #1: Four (4) replicates of each human urine sample (50.0, 75.0, 100.0, 150.0, 200.0, 250.0, 500.0, 1000.0, 1500.0, 2500.0 ng/mL etizolam) were tested.

Run #2: Run #1 was repeated.

A total of 40 replicates per precision sample were tested. Mean determinations of the ARK™ HS Benzodiazepine II Assay, standard deviation (SD) for the within-run, between-day, and total coefficients of variation (% CVs) were calculated.

Expected Performance: Total Precision ≤12% CV for concentrations ≥50 ng/mL and SD <15 for concentrations <50 ng/mL.

##### Spike Recovery

The precision data for spike recovery were analyzed using the following equation:

$$\%\mathrm{Recovery}\;=\frac{\mathrm{Mean}\;\mathrm{recovered}\;\mathrm{concentration}}{\mathrm{Theoretical}\;\mathrm{concentration}}\times 100$$

Expected Performance: analytical recovery within ±15% of the expected level for concentrations ≥50 ng/mL and ± 15 ng/mL for concentrations <50 ng/mL.

### 4.4. Sample Analysis Protocol

Urine samples (N = 501) were collected from patients under withdrawal treatments, after informed consent, and from postmortem intoxication cases. The samples were anonymized after collection and stored at −20 °C until analysis. One aliquot was processed without sample pretreatment, while 50 µL β-glucuronidase was added to another aliquot that was kept at 37 °C in the dark for 16 h, before injection in the Siemens ADVIA^®^ 1800 Clinical Chemistry System. A total of 1002 samples were analyzed through the two immunoassays and the LC-MS/MS method. Urine samples were analyzed through the LC-MS/MS system after the following sample treatment: 100 µL of urine were put into a glass tube containing 200 µL of borate buffer solution (pH 9) and deuterated internal standards. Then, samples were vortexed after the addition of 1.5 mL of hexane:ethyl acetate (7:3) mixture solution and centrifuged at 4000 rpm for 5 min. The supernatant was separated and evaporated to dryness under a nitrogen stream at 75 °C. Samples were reconstituted in 200 µL of mobile phase; finally, 5 µL were injected into the LC-MS/MS system. MRM transitions chromatograms of all the monitored substances in a spiked urine sample are reported as Supplementary Material.

The sensitivity was expressed by the following equation:

$$\mathrm{Sensitivity}\;\left(\mathrm{SE}\right)=\frac{\mathrm{True}\;\mathrm{Positives}\;\left(\mathrm{TP}\right)}{\mathrm{True}\;\mathrm{Positives}\;\left(\mathrm{TP}\right)+\mathrm{False}\;\mathrm{Negatives}\;\left(\mathrm{FN}\right)}$$

Specificity was expressed by the following equation

$$\mathrm{Specificity}\;\left(\mathrm{SP}\right)=\frac{\mathrm{True}\;\mathrm{Negatives}\;\left(\mathrm{TN}\right)}{\mathrm{True}\;\mathrm{Negatives}\;\left(\mathrm{TN}\right)+\mathrm{False}\;\mathrm{Positives}\;\left(\mathrm{FP}\right)}$$

Cross reactivity was calculated on the basis of the following equation

$$\mathrm{Cross}\;\mathrm{reactivity}\;\left(\%\right)=\frac{\mathrm{Measured}\;\mathrm{concentration}}{\mathrm{Spiked}\;\mathrm{concentration}}\times 100$$

## 5. Conclusions

This study confirmed the need for a hydrolysis pretreatment in order to increase the sensitivity of the methods in identifying benzodiazepines and metabolites in urine. The immunoassay methods provided positive results for most of the new designer benzodiazepines spiked in urine samples and in an authentic positive case. A high specificity was observed for both immunoassays. Furthermore, the ARK™ HS Benzodiazepine II Assay provided better results in term of sensitivity. In particular, an extremely high signal was observed in urine samples that tested positive for lorazepam and 7-aminoclonazepam. Indeed, the new immunoassay was demonstrated to be a reliable screening tool for monitoring a recent consumption of lorazepam, by guaranteeing the detection of this benzodiazepine at concentrations lower than 1 ng/mL without the need for sample hydrolysis. Hence, the ARK™ HS Benzodiazepine II Assay proved to be a promising reliable screening method for benzodiazepines in urine. The new kit could now be added to the routine workflow of laboratories, and, in the future, it should be compared to other available immunoassays in order to evaluate its performance.

## Figures and Tables

**Table 1:** LC-MS/MS results from urine samples.

Molecule	Urine	No. Positive Cases	Min-Max (ng/mL)	Mean (ng/mL)	Median (ng/mL)
Oxazepam	AH *	45	42.4–25,300.0	4734.6	2620.0
	NH *	24	54.7–3260.0	357.1	169.5
Nordazepam	AH *	38	33.4–13,000.0	1820.6	669.0
	NH *	34	14.5–2080.0	335.0	105.0
Desalkylflurazepam	AH *	35	10.0–435.0	102.1	61.6
	NH *	15	12.1–125.0	65.1	57.1
Diazepam	AH *	26	10.0–315.0	86.3	72.2
	NH *	25	10.0–566.0	109.8	56.5
Alprazolam	AH *	21	12.6–1850.0	527.9	349.0
	NH *	17	14.7–480.0	143.1	105.0
Delorazepam	AH *	21	10.6–282.0	96.3	91.2
	NH *	12	22.0–239.0	110.1	83.5
Lorazepam	AH *	19	28.2–20,500.0	2235.6	1100.0
	NH *	6	11.1–1030.0	381.1	116.2
2-hydroxyethylflurazepam	AH *	14	94.7–127,000.0	27,561.9	7715.0
	NH *	10	66.8–6110.0	2211.3	1540.0
7-aminoclonazepam	AH *	13	11.9–198.0	75.9	72.5
	NH *	8	15.6–135.0	77.9	81.9
Flurazepam	AH *	11	12.3–1130.0	261.9	116.0
	NH *	14	11.7–1070.0	222.8	125.0
α-hydroxyalprazolam	AH *	6	69.8–582.0	217.9	127.5
	NH *	0	-	-	-
Lormetazepam	AH *	4	67.2–482.0	310.6	346.5
	NH *	0	-	-	-
Bromazepam	AH *	3	137.0–2650.0	1261.7	998.0
	NH *	3	129.0–3010.0	1290.0	731.0
7-aminoflunitrazepam	AH *	3	10.2–13.5	12.2	12.9
	NH *	0	-	-	-
Triazolam	AH *	2	21.6–791.0	-	-
	NH *	2	27.7–332.0	-	-
Flubromazepam	AH *	1	330.0	-	-
	NH *	1	194.0	-	-

* NH: non-hydrolyzed; AH: after hydrolysis.

**Table 2:** Diagnostic sensitivity and specificity of the two immunoassays (LC-MS/MS data used as gold standard).

Cut-Off (ng/mL)	Urine	EMIT^®^ II PLUS Benzodiazepine Assay						ARK™ HS Benzodiazepine II Assay					
		TN * (N	FP * (N)	TP * (N)	FN * (N)	SE * $\frac{\left(TP\right)}{\left(TP\right)+\left(FN\right)}$	SP * $\frac{\left(TN\right)}{\left(TN\right)+\left(FP\right)}$	TN * (N)	FP * (N)	TP * (N)	FN * (N)	SE * $\frac{\left(TP\right)}{\left(TP\right)+\left(FN\right)}$	SP * $\frac{\left(TN\right)}{\left(TN\right)+\left(FP\right)}$
50	NH *	401	0	89	11	0.89	1.00	396	5	98	2	0.98	0.99
	AH *	398	3	94	6	0.94	0.99	396	5	100	0	1.00	0.99
100	NH *	401	0	74	26	0.74	1.00	396	5	97	3	0.97	0.99
	AH *	398	3	88	12	0.88	0.99	396	5	98	2	0.98	0.99
200	NH *	401	0	64	36	0.64	1.00	396	5	91	9	0.91	0.99
	AH *	399	2	79	21	0.79	0.99	396	5	92	8	0.92	0.99

* NH: non-hydrolyzed; AH: after hydrolysis; TN: true negative; FP: false positive; TP: true positive; FN: false negative; SE: diagnostic sensitivity; SP: diagnostic specificity.

**Table 3:** Cross reactivity of designer benzodiazepines measured by the two immunoassays.

Molecule	% Cross-Reactivity EMIT^®^ II PLUS Benzodiazepine Assay (Calibrated with Lormetazepam)	% Cross-Reactivity ARK™ HS Benzodiazepine II Assay (Developed on Etizolam)
Bentazepam	40	15
Clonazolam	100	40
Diclazepam	>1000	>1000
Etizolam	60	100
Flualprazolam	300	650
Flubromazolam	300	400
Phenazepam	200	>1000

**Table 4:** LODs and LOQs of the monitored substances by LC-MS/MS method.

Substance	LOD (ng/mL)	LOQ (ng/mL)	Substance	LOD (ng/mL)	LOQ (ng/mL)
α-hydroxyalprazolam	2.5	8.5	Flualprazolam	0.3	1.0
2-hydroxyethylflurazepam	3.7	12.3	Flubromazepam	1.1	3.8
7-aminoclonazepam	0.6	1.9	Flubromazolam	0.4	1.2
7-aminoflunitrazepam	0.8	2.6	Flurazepam	0.4	1.4
Alprazolam	0.8	2.7	Lorazepam	3.0	9.9
Bentazepam	5.0	16.7	Lormetazepam	0.1	0.3
Bromazepam	4.0	13.3	Midazolam	0.7	2.4
Clonazolam	0.5	1.6	Nordiazepam	0.2	0.7
Delorazepam	0.6	2.0	Oxazepam	0.5	1.7
Desalkylflurazepam	0.4	1.4	Phenazepam	1.0	3.3
Diazepam	0.3	0.9	Temazepam	1.0	3.6
Diclazepam	0.3	0.9	Triazolam	1.1	3.8
Etizolam	0.2	0.5			

## Data Availability

Data sharing not applicable.

## Supplementary Materials

The following are available online, Ion extracted chromatograms of all the monitored substances in a spiked urine sample. Figure S1: Chromatograms of motiored substances in spiked urine.

## Sample Availability

Samples of the compounds are available from the authors.
