# Supplementary figures and images for: Comparison of Two Immunoassay Screening Methods and a LC-MS/MS in Detecting Traditional and Designer Benzodiazepines in Urine
Source: Molecules. 2021 Dec 24;27(1):112. doi: 10.3390/molecules27010112 (PMC8746686; doi:10.3390/molecules27010112)

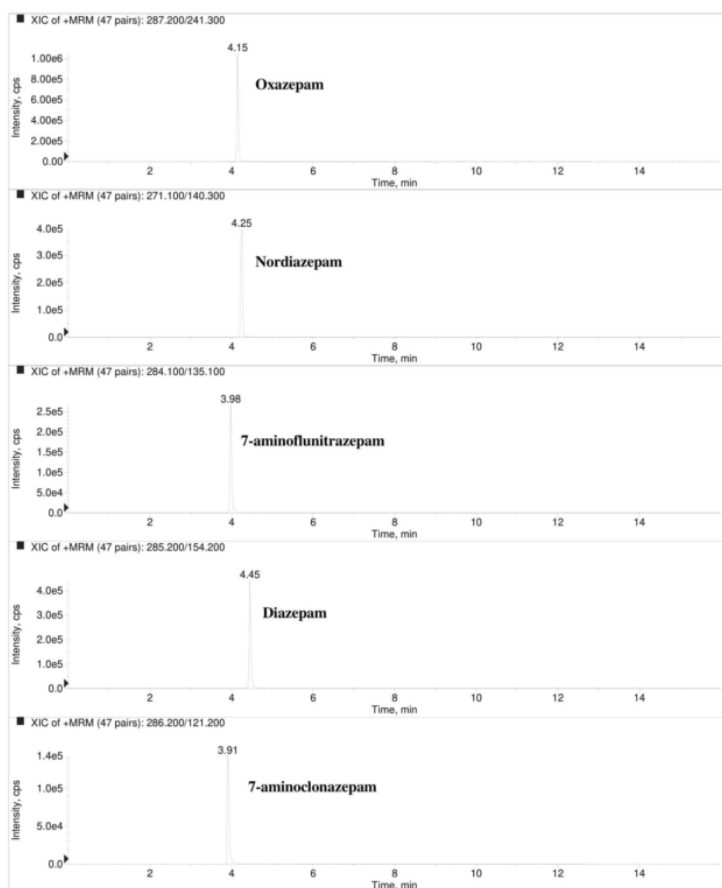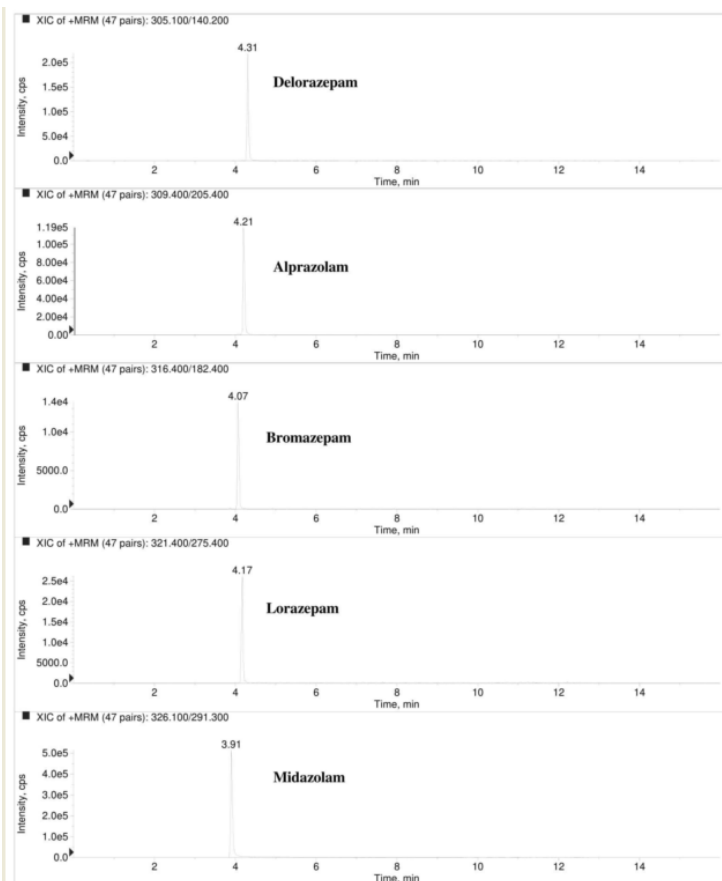

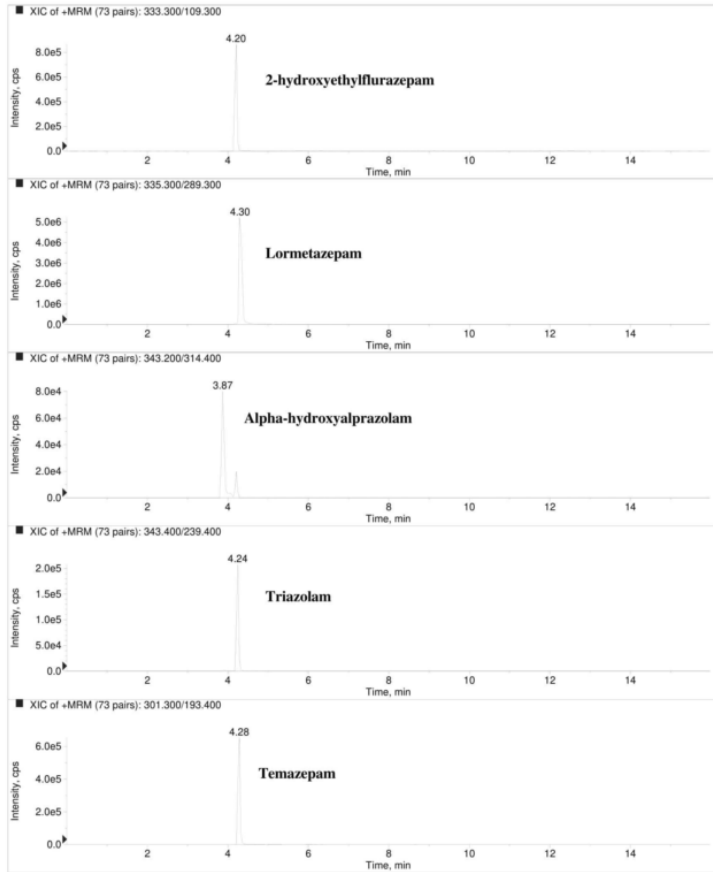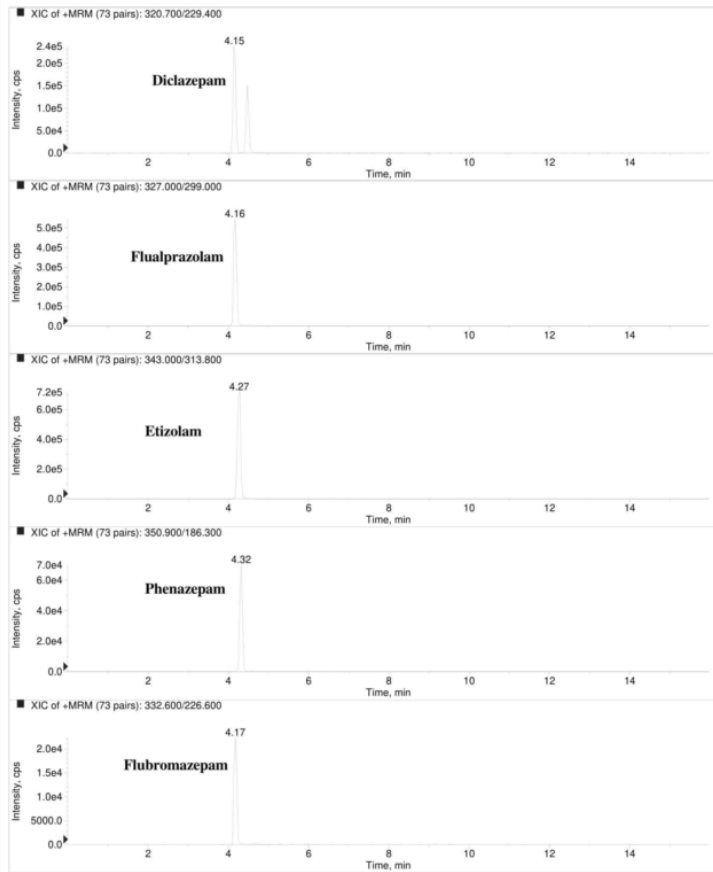

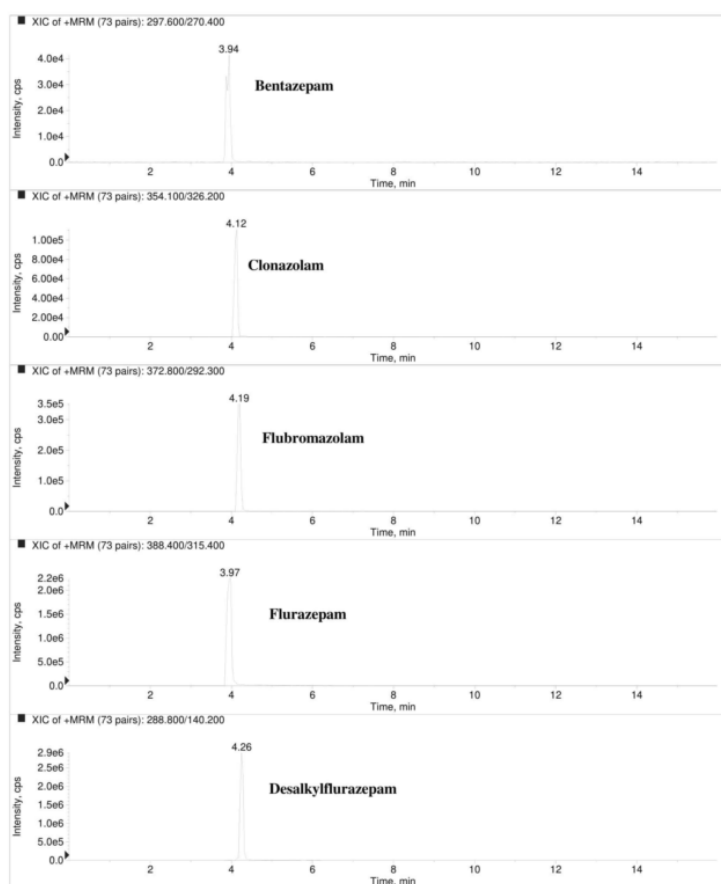

Figure S1. Chromatograms of monitored substances in spiked urine.

Supplement: Supplementary file 1 [file molecules-27-00112-s001.zip › molecules-1495637-SI.pdf]
